# Association Between Hypovitaminosis D and Angiographic Severity of Coronary Artery Disease in Bangladesh

**DOI:** 10.7759/cureus.115998

**Published:** 2026-09-09

**Authors:** Md. Maruf-Ur- Rahman, Md. Rafiqul Islam, Wazeda Begum, Md. Fakhrul Islam Khaled, A. H. M. Golam Kibria, Chaudhury Meshkat Ahmed

**Affiliations:** 1 Biochemistry, Dhaka National Medical College, Dhaka, BGD; 2 Biochemistry and Molecular Biology, Jagannath University, Dhaka, BGD; 3 Dermatology and Venereology, Dhaka National Medical College, Dhaka, BGD; 4 Cardiology, Bangladesh Medical University, Dhaka, BGD; 5 Epidemiology and Biostatistics, Centre for Medical Research and Development, Dhaka, BGD

**Keywords:** coronary angiography, coronary artery disease, coronary artery disease severity, hypovitaminosis d, multivessel disease, vitamin d deficiency

## Abstract

Background

Coronary artery disease (CAD) remains a major cause of morbidity and mortality worldwide. Vitamin D deficiency has been increasingly linked to the pathophysiology of atherosclerosis; however, its association with the severity of CAD remains inconclusive, particularly in Bangladeshi populations where hypovitaminosis D is highly prevalent. The primary objective of this study was to compare serum vitamin D status between patients with angiographically confirmed CAD and those without CAD. The secondary objective was to evaluate the association between serum vitamin D levels and the angiographic severity of CAD and to explore the ability of vitamin D to discriminate multivessel from single-vessel disease among patients with angiographically confirmed CAD.

Materials and methods

This hospital-based cross-sectional comparative study was conducted among 300 adults undergoing coronary angiography at Bangladesh Medical University and Dhaka National Medical Institute Hospital from January to December 2024. Eligible participants were screened consecutively and enrolled within predefined angiographic strata until 150 participants with angiographically confirmed CAD and 150 participants without angiographic evidence of CAD had been included. Sociodemographic, clinical, anthropometric, biochemical, and angiographic variables were assessed contemporaneously and systematically recorded using a structured case record form. Serum vitamin D, fasting lipid profile, HbA1c, calcium, alkaline phosphatase, and parathyroid hormone levels were measured using standard laboratory methods. Among participants with CAD, angiographic severity was assessed according to the extent of coronary vessel involvement.

Results

Mean serum vitamin D levels were significantly lower among participants with CAD than among those without CAD (18.78 ± 6.66 ng/mL vs. 29.25 ± 3.84 ng/mL; p < 0.001). Triple-vessel disease was the most frequent angiographic pattern, observed in 68 (45.3%) patients with CAD. Serum vitamin D levels showed a significant inverse correlation with the extent of coronary vessel involvement (rₛ = -0.262, p = 0.001), indicating that lower vitamin D levels were associated with greater angiographic severity. Exploratory ROC analysis identified a serum vitamin D threshold of ≤21.54 ng/mL for discriminating multivessel from single-vessel CAD, with an area under the curve of 0.619 (95% CI: 0.513-0.725; p = 0.025), sensitivity of 72.5%, specificity of 53.7%, and overall accuracy of 67.3%, indicating limited discriminative ability. In multivariable logistic regression analysis, age <50 years (AOR = 3.614, 95% CI: 1.380-9.467; p = 0.009) and serum vitamin D ≤21.54 ng/mL (AOR = 2.669, 95% CI: 1.241-5.741; p = 0.012) were independently associated with multivessel CAD within the study population.

Conclusions

Hypovitaminosis D was significantly associated with greater angiographic severity and multivessel involvement among patients with CAD. Although a serum vitamin D threshold of ≤21.54 ng/mL demonstrated limited discriminatory performance for multivessel CAD, lower vitamin D levels remained independently associated with more extensive coronary involvement after multivariable adjustment. These findings support a potential role for serum vitamin D as an adjunctive marker of CAD severity, although its clinical utility requires further validation in larger prospective studies.

## Introduction

Coronary artery disease (CAD) is a leading cause of death globally and a significant cause of disability and healthcare burden [1]. South Asian populations, including Bangladeshis, demonstrate a disproportionately higher susceptibility to CAD. In these populations, the disease frequently manifests at a younger age, progresses more aggressively, and is often associated with more extensive angiographic involvement compared with other ethnic groups [2]. Reliable nationwide estimates of CAD prevalence in Bangladesh are limited. More recent studies suggest that the prevalence of CAD ranges from 1.85% to 3.4% in rural areas and from 19.6% to 20.6% among employed individuals in urban areas [3,4]. Despite the variation in reported estimates, available evidence suggests that both the burden and severity of CAD are increasing in Bangladesh.

The rising prevalence and clinical severity of CAD have underscored the need for continued investigation into additional factors implicated in its pathogenesis and progression. Several conventional risk factors for CAD, such as hypertension, dyslipidemia, smoking, diabetes mellitus, and genetic predisposition, have been firmly established through extensive epidemiological and clinical research. Nevertheless, these traditional risk factors are not universally present among all patients with CAD, suggesting that other underlying mechanisms may contribute to the initiation and progression of atherosclerosis [5].

According to a report from the American College of Cardiology, new risk factors for cardiovascular diseases have been identified in recent years. These include factors such as the gut microbiota, homocysteine and vitamin D levels, sleep apnea, sleep duration, plasma uric acid concentration, airborne contaminants, and climate change [6]. Consequently, identifying novel and potentially modifiable risk factors has become an important area of cardiovascular research because these factors may offer new opportunities for risk stratification, prevention, and therapeutic intervention. In recent years, vitamin D deficiency (VDD) has emerged as a potential contributor to the development and progression of atherosclerotic cardiovascular disease [7]. Vitamin D may also influence several key mechanisms involved in atherogenesis through its regulatory effects on lipid metabolism, macrophage activity, endothelial function, and vascular inflammation. In particular, vitamin D has been reported to inhibit cholesterol uptake by macrophages. In the presence of VDD, this protective regulatory effect may be diminished, thereby promoting cholesterol accumulation within macrophages and leading to the formation of lipid-laden foam cells. These foam cells may infiltrate the vascular intima, contribute to atheromatous plaque formation, and accelerate atherosclerotic progression [8].

Epidemiological data reveal a worldwide pandemic of VDD, which persisted from 2000 to 2022 [9]. The pooled prevalence of VDD in South Asia has been reported to be 68%. The highest prevalence was reported in Pakistan (73%), followed by Bangladesh (67%), India, Nepal, and Sri Lanka. Women had a higher prevalence of VDD than men [10]. Sahani and Gupta (2024) reported a significant negative correlation between blood vitamin D levels and both the Gensini and SYNTAX scores, indicating that lower vitamin D levels were associated with greater angiographic severity of CAD [11]. On the other hand, Alsancak et al. found no significant association between serum vitamin D levels and the degree and extent of CAD [12]. Although several observational studies have shown a significant negative correlation between serum vitamin D levels and the severity of coronary stenotic lesions, the literature remains inconsistent [11,12].

Given the inconsistent evidence regarding the relationship between vitamin D status and CAD, further investigation is warranted, particularly in resource-limited settings where data on angiographically assessed disease severity remain limited. Evidence from Bangladesh specifically examining the association between serum vitamin D levels and the severity of coronary involvement is scarce. Therefore, the primary objective of this study was to compare serum vitamin D status between patients with angiographically confirmed CAD and those without angiographic evidence of CAD. The secondary objective was to evaluate the association between serum vitamin D levels and the angiographic severity of CAD and to explore the ability of serum vitamin D to discriminate between multivessel and single-vessel disease among patients with angiographically confirmed CAD.

## Materials and methods

Study design, setting, and ethical considerations

This hospital-based cross-sectional comparative study was conducted in collaboration with the Department of Cardiology at Bangladesh Medical University and Dhaka National Medical Institute Hospital, Dhaka, Bangladesh, from January to December 2024. The study protocol was approved by the Institutional Ethical Review Board of Dhaka National Medical College (approval number: DNMC/IERB/Ethical/2023/2027). Written informed consent was obtained from all participants before enrollment. Confidentiality and anonymity of participant information were maintained throughout the study in accordance with the principles of the Declaration of Helsinki.

Sample size calculation

The sample size was calculated using the formula for comparison of two independent proportions. Based on a previous study, VDD was observed in 95.7% of patients with CAD and 80.2% of those without CAD [13]. Considering a two-sided significance level of 1% (α = 0.01), 90% power (β = 0.10), and an equal allocation ratio of 1:1, the minimum required sample size was 129 participants in each group (total 258). To improve the precision of the study estimates and allow for potential incomplete data or exclusions, 150 participants were enrolled in each group, resulting in a final sample size of 300.

Participant recruitment

Adult patients undergoing diagnostic coronary angiography for clinically suspected CAD were recruited from the inpatient cardiology units, outpatient cardiology services, and emergency department [14]. Men and women aged 18-70 years who had been referred for coronary angiography and fulfilled the prespecified eligibility criteria were considered for inclusion.

Eligible patients were screened consecutively during the study period. Following coronary angiography, participants were classified into two predefined angiographic strata: those with angiographically confirmed CAD and those without angiographic evidence of CAD. Enrollment continued within each stratum until the prespecified sample size of 150 participants per group was achieved. Thus, the final study population comprised 150 participants with CAD and 150 participants without CAD. The equal allocation was predetermined to facilitate comparison between the two angiographic groups and was not intended to represent the natural prevalence of CAD among patients undergoing coronary angiography. Accordingly, the fixed 1:1 CAD-to-non-CAD ratio reflects the study sampling framework rather than the distribution of CAD in the source population. All participants underwent coronary angiography for a clinical indication following evaluation for suspected CAD within the participating cardiology services. Therefore, the non-CAD group did not represent healthy individuals from the general population; rather, it consisted of clinically evaluated patients with coronary angiography showing no CAD. Both groups originated from the same clinical referral setting and were subsequently classified based on their angiographic findings.

CAD was operationally defined as >50% luminal diameter narrowing in at least one major epicardial coronary artery [15]. Participants meeting this angiographic criterion were classified in the CAD group, whereas those without angiographic evidence of CAD were classified in the non-CAD group. Among participants with CAD, angiographic severity was determined according to the number of major coronary vessels involved. Participants were initially classified as having single-, double-, or triple-vessel disease. For subsequent analyses, double- and triple-vessel disease were combined and categorized as multivessel CAD. Coronary angiograms were independently assessed and confirmed by experienced cardiologists to ensure consistency of angiographic classification. For both groups, participants were excluded if they had conditions or treatments likely to substantially influence serum vitamin D or calcium-parathyroid hormone homeostasis, including chronic kidney disease, chronic liver disease, parathyroid dysfunction, chronic inflammatory disorders, malignancy, pregnancy, recent significant infection, or current use of vitamin D or calcium supplementation.

Data collection and study measurements

Data were collected using a structured form that had been pretested prior to formal data collection. Sociodemographic information included age, sex, residential area, educational level, and socioeconomic status. Clinical variables included smoking status, body mass index (BMI), diabetes mellitus, hypertension, and family history of CAD [14].

For participants with angiographically confirmed CAD, the number of affected coronary vessels and the extent of vessel involvement were recorded. The principal biochemical variables assessed were glycated hemoglobin (HbA1c), fasting lipid profile, serum 25-hydroxyvitamin D, total serum calcium, alkaline phosphatase (ALP), and parathyroid hormone.

Blood collection and laboratory procedures

Following a fasting period of at least 10 hours, venous blood samples were obtained under sterile conditions. Approximately 3.0 mL of blood was placed into a sterile tube containing ethylenediaminetetraacetic acid for the necessary whole-blood investigations [14]. An additional 3.0 mL of blood was collected in a plain vacuum tube for serum biochemical analysis.

Serum was separated by centrifugation, and, whenever feasible, laboratory investigations were performed on the same day. When immediate analysis could not be performed, the serum samples were temporarily stored at 2-10°C until processing [14].

HbA1c, fasting lipid parameters, serum 25-hydroxyvitamin D, calcium, ALP, and parathyroid hormone were analyzed using established laboratory methods and automated platforms. The analytical systems included the VITROS® 5600 Integrated System (Ortho Clinical Diagnostics, Raritan, NJ, USA), Bio-Rad D-10 HbA1c Analyzer, Atellica CH 930 Chemistry Analyzer (Siemens Healthineers), and ADVIA Centaur XP Immunoassay System (Siemens Healthineers, Tarrytown, NY, USA) [14]. Low-density lipoprotein (LDL) was calculated from fasting total cholesterol, high-density lipoprotein (HDL), and triglyceride concentrations using the Friedewald equation [16]:

$\mathrm{LDL\mathrm{-}C}\,(\mathrm{mg/dL}) = \text{total cholesterol} - \mathrm{HDL\mathrm{-}C} - \frac{\mathrm{triglycerides}}{5}$

All tests were conducted according to the manufacturers’ operating instructions and the routine internal quality-control procedures of the respective laboratories.

Measurement and categorization of vitamin D

Serum vitamin D was measured using the Abbott ARCHITECT ci4100 analyzer (Abbott, Max-Planck Ring 2, Germany). The assay employed a competitive immunoassay to quantitatively determine circulating vitamin D, with an analytical range of 4.0-160.0 ng/mL. Vitamin D status was categorized as severe deficiency (<10 ng/mL), deficiency (10 to <20 ng/mL), insufficiency (20 to <30 ng/mL), and sufficiency (30-100 ng/mL), based on thresholds previously applied in a Bangladeshi population [17].

Statistical analysis

Statistical analyses were conducted using SPSS Statistics version 26.0 (IBM Corp. Released 2019. IBM SPSS Statistics for Windows, Version 26.0. Armonk, NY). The distribution of continuous variables was assessed using the Shapiro-Wilk test. As most continuous variables were non-normally distributed, they were primarily summarized as median (range), with mean ± standard deviation (SD) also reported where appropriate. Categorical variables were presented as frequency and percentage (n (%)). Categorical variables were compared between the CAD and non-CAD groups using the chi-square test. The Mann-Whitney U test was used to compare continuous variables between two independent groups.

In contrast, the Kruskal-Wallis test was used for comparisons involving three groups, followed by pairwise post hoc comparisons where appropriate. The association between serum vitamin D levels and the extent of coronary vessel involvement was assessed using Spearman’s rank correlation coefficient. Receiver operating characteristic (ROC) curve analysis was performed among participants with angiographically confirmed CAD to assess the discriminatory ability of serum vitamin D levels to distinguish multivessel from single-vessel CAD. The exploratory threshold was determined using the Youden index. Sensitivity, specificity, positive predictive value, negative predictive value, and overall accuracy were subsequently calculated with their corresponding 95% confidence intervals (CIs). Variables that showed statistically significant associations with multivessel CAD in the initial analysis, namely age, serum vitamin D level ≤21.54 ng/mL, and obesity, were evaluated using univariable logistic regression. Variables that remained statistically significant in the univariable analysis, namely age <50 years and serum vitamin D level ≤21.54 ng/mL, were subsequently entered into the multivariable logistic regression model to assess their independent associations with multivessel CAD. Accordingly, the estimates for age and vitamin D level were mutually adjusted. Obesity was not included in the final multivariable model because it did not reach statistical significance in the univariable analysis (p = 0.056). Results were reported as odds ratios (ORs) with corresponding 95% CIs. All statistical tests were two-tailed, and a p < 0.05 was considered statistically significant.

## Results

Most participants in both groups were aged ≥50 years, with comparable age distributions and median ages between the CAD and non-CAD groups (p = 0.401). In contrast, male participants were significantly more common in the CAD group than in the non-CAD group (114 (76.0%) vs. 95 (63.3%); p = 0.017). Participants in the CAD group also had a significantly higher median BMI than those in the non-CAD group (27.25 vs. 25.70 kg/m²; p < 0.001) (Table 1).

**Table 1:** Demographic characteristics of the study participants (N = 300) ^a^Chi-square test; ^b^Mann-Whitney U test. Data are presented as frequencies (%) and medians (range). BMI: body mass index, CAD: coronary artery disease

Demographic characteristics		Participants		Total	p-value
		CAD	Non-CAD		
		n = 150	n = 150		
Age (years)	<50	51 (34.0)	58 (38.7)	109 (36.3)	0.401ᵃ
	≥50	99 (66.0)	92 (61.3)	191 (63.7)	
	Median (range)	52 (19-68)	52 (28-70)	52 (19-70)	0.569ᵇ
Gender	Male	114 (76.0)	95 (63.3)	209 (69.7)	0.017ᵃ
	Female	36 (24.0)	55 (36.7)	91 (30.3)	
BMI	Median (range)	27.25 (23.20-33.30)	25.70 (22.90-32.60)	26.5 (22.90-33.30)	<0.001ᵇ

The CAD group had significantly higher total cholesterol (221.75 ± 39.03 mg/dL vs. 136.91 ± 13.84 mg/dL; p < 0.001), triglyceride (260.62 ± 45.47 mg/dL vs. 136.72 ± 13.89 mg/dL; p < 0.001), LDL (140.81 ± 38.14 mg/dL vs. 67.59 ± 13.87 mg/dL; p < 0.001), and HbA1c levels (6.86 ± 1.49% vs. 5.45 ± 0.53%; p < 0.001) than the non-CAD group. In contrast, HDL cholesterol was significantly lower in the CAD group (28.82 ± 3.92 mg/dL vs. 41.97 ± 4.39 mg/dL; p < 0.001). However, ALP levels did not differ significantly between the groups (p = 0.136). Regarding vitamin D status, deficiency was most common among participants in the CAD group (77 (51.3%)), whereas insufficiency was most common in the non-CAD group (88 (58.7%)). Mean serum vitamin D level was significantly lower in the CAD group (18.78 ± 6.66 ng/mL vs. 29.25 ± 3.84 ng/mL; p < 0.001). The CAD group also had significantly lower calcium levels (9.09 ± 0.44 mg/dL vs. 9.40 ± 0.46 mg/dL; p < 0.001) and higher parathyroid hormone (PTH) levels (76.60 ± 32.82 pg/mL vs. 60.87 ± 43.24 pg/mL; p < 0.001) than the non-CAD group (Table 2).

**Table 2:** Comparison of serum lipid profile, vitamin D, HbA1c, calcium, and parathyroid hormone between the CAD and non-CAD groups (N = 300) ^a^Mann-Whitney U test; ^b^Chi-square test. Data are presented as mean ± SD, frequencies (percentage), and medians (range). HDL: high-density lipoprotein, LDL: low-density lipoprotein, HbA1c: glycated hemoglobin, ALP: alkaline phosphatase, PTH: parathyroid hormone, CAD: coronary artery disease, SD: standard deviation

Laboratory findings		Participants		Total	p-value
		CAD	Non-CAD		
		n = 150	n = 150		
Total cholesterol (mg/dL)	Mean ± SD	221.75 ± 39.03	136.91 ± 13.84	179.33 ± 51.58	<0.001^a^
	Median (range)	224.50 (121.00-388.00)	135.50 (110.00-167.00)	159.50 (110.00-388.00)	
HDL (mg/dL)	Mean ± SD	28.82 ± 3.92	41.97 ± 4.39	35.40 ± 7.79	<0.001^a^
	Median (range)	30.00 (19.00-35.00)	41.00 (33.00-55.00)	35.00 (19.00-55.00)	
LDL (mg/dL)	Mean ± SD	140.81 ± 38.14	67.59 ± 13.87	104.20 ± 46.54	<0.001^a^
	Median (range)	143.4 (46.80-314.80)	67.00 (30.60-101.40)	87.00 (30.60-314.80)	
Triglyceride (mg/dL)	Mean ± SD	260.62 ± 45.47	136.72 ± 13.89	198.67 ± 70.55	<0.001^a^
	Median (range)	256.00 (141.00-374.00)	137.00 (110.00-161.00)	160.00 (110.00-374.00)	
Vitamin D (ng/mL)	Severe deficiency	15 (10.0)	0 (0.0)	15 (5.0)	<0.001^b^
	Deficiency	77 (51.3)	0 (0.0)	77 (25.7)	
	Insufficiency	50 (33.3)	88 (58.7)	138 (46.0)	
	Sufficiency	8 (5.3)	62 (41.3)	70 (23.3)	
	Mean ± SD	18.78 ± 6.66	29.25 ± 3.84	24.00 ± 7.55	<0.001^a^
	Median (range)	18.5 (4.94-38.44)	29.14 (20.22-39.20)	25.82 (4.94-39.20)	
HbA1c (%)	Mean ± SD	6.86 ± 1.49	5.45 ± 0.53	6.15 ± 1.32	<0.001^a^
	Median (range)	6.30 (5.10-11.90)	5.50 (4.50-6.30)	5.85 (4.50-11.90)	
Calcium (mg/dL)	Mean ± SD	9.09 ± 0.44	9.40 ± 0.46	9.25 ± 0.47	<0.001^a^
	Median (range)	9.10 (7.80-10.00)	9.40 (8.50-10.30)	9.20 (7.80-10.30)	
ALP (U/L)	Mean ± SD	85.15 ± 20.96	86.67 ± 17.28	85.91 ± 19.19	0.136^a^
	Median (range)	83.00 (54.00-165.00)	87.00 (55.00-120.00)	85.50 (54.00-165.00)	
PTH (pg/mL)	Mean ± SD	76.60 ± 32.82	60.87 ± 43.24	68.74 ± 39.12	<0.001^a^
	Median (range)	65.30 (29.80-187.00)	55.00 (27.20-351.00)	61.90 (27.20-351.00)	

Among participants in the CAD group, ST-elevation myocardial infarction (STEMI) was the most common clinical presentation (64 (42.7%)), followed by chronic stable angina (CSA) (39 (26.0%)), unstable angina (UA) (30 (20.0%)), and non-STEMI (NSTEMI) (17 (11.3%)). Regarding disease severity, triple-vessel disease was the most frequent angiographic finding (68 (45.3%)), whereas single-vessel disease and double-vessel disease were each observed in 41 (27.3%) participants. Hypertension was the most common comorbidity (55 (36.7%)), followed by diabetes mellitus (45 (30.0%)). Smoking was reported in 20 (13.3%) participants, obesity in 18 (12.0%), and a family history of CAD in 12 (8.0%) (Figure 1).

**Figure 1:**
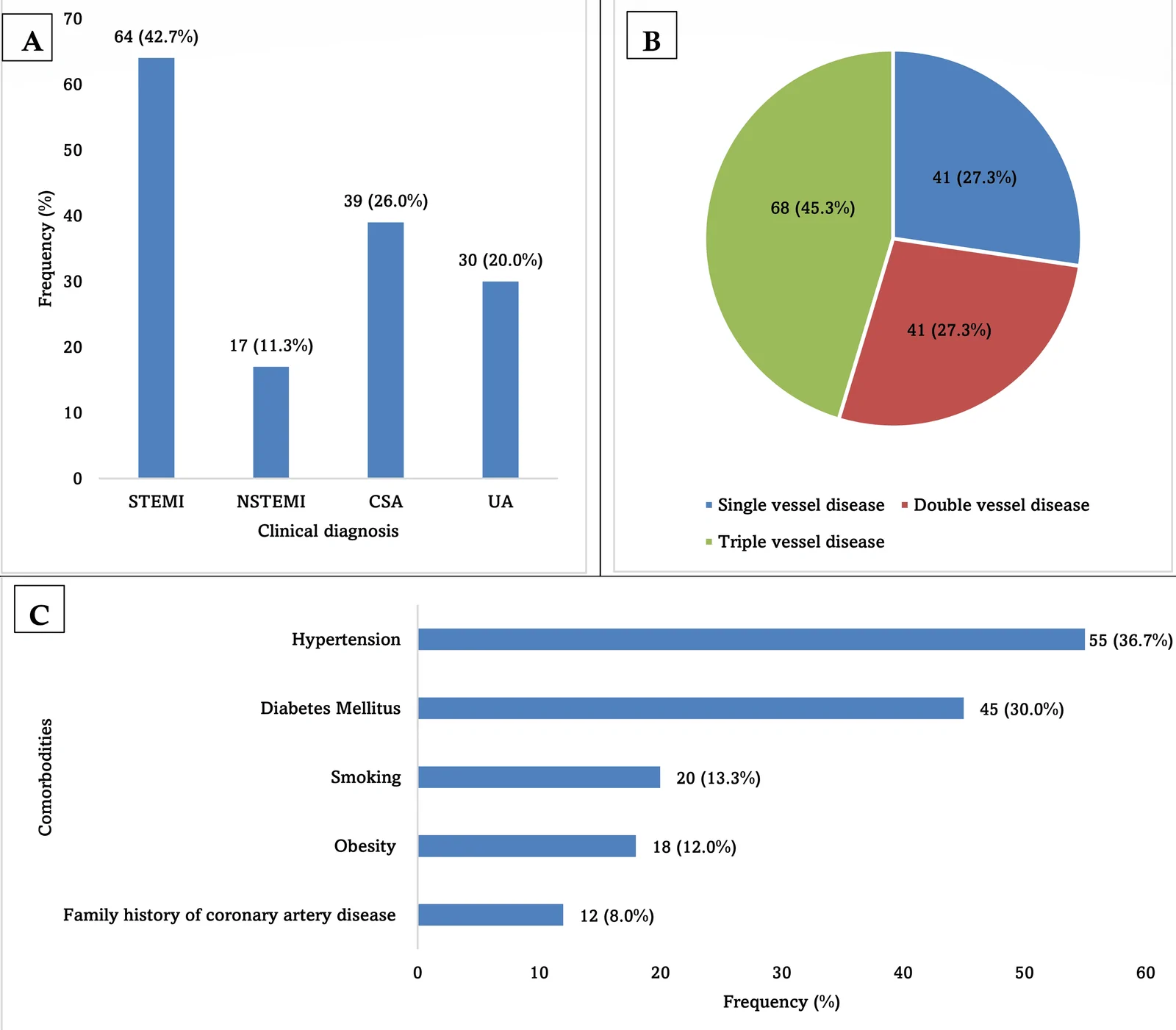
Distribution of participants in the CAD group according to (A) clinical diagnosis, (B) severity of CAD, and (C) comorbidities STEMI: ST-elevation myocardial infarction, NSTEMI: non-ST-elevation myocardial infarction, CSA: chronic stable angina, UA: unstable angina, CAD: coronary artery disease

The mean vitamin D level was highest among participants with single-vessel disease (21.10 ± 7.59 ng/mL), followed by those with double-vessel disease (19.38 ± 6.05 ng/mL), and was lowest among those with triple-vessel disease (16.98 ± 5.95 ng/mL). A similar pattern was observed for the median vitamin D levels. The overall difference among the three groups was statistically significant (p = 0.014). In pairwise comparisons, the difference between single-vessel and triple-vessel disease was statistically significant (p = 0.016). In contrast, the differences between single-vessel and double-vessel disease (p > 0.99) and between double-vessel and triple-vessel disease (p = 0.175) were not statistically significant. These findings indicate that lower serum vitamin D levels were associated with greater coronary vessel involvement (Table 3).

**Table 3:** Comparison of serum vitamin D levels according to the extent of coronary vessel involvement among participants with CAD (n = 150) ^a^Kruskal-Wallis test and pairwise comparison (x, y, z); ^x^single-vessel disease vs. double-vessel disease; ^y^double-vessel disease vs. triple-vessel disease; and ^z^single-vessel disease vs. triple-vessel disease. Data are presented as mean ± SD and median (range). CAD: coronary artery disease, SD: standard deviation

Laboratory findings		CAD severity			p-value
		Single-vessel disease	Double-vessel disease	Triple-vessel disease	
Vitamin D (ng/mL)	Mean ± SD	21.10 ± 7.59	19.38 ± 6.05	16.98 ± 5.95	0.014^a^
	Median (range)	21.70 (8.36-38.44)	18.77 (4.94-36.11)	15.48 (7.69-31.40)	>0.99^x^
					0.175^y^
					0.016^z^

A weak but statistically significant negative correlation was observed between serum vitamin D levels and the extent of coronary vessel involvement (rₛ = -0.262, p = 0.001), indicating that lower vitamin D levels were associated with involvement of a greater number of coronary vessels (Figure 2).

**Figure 2:**
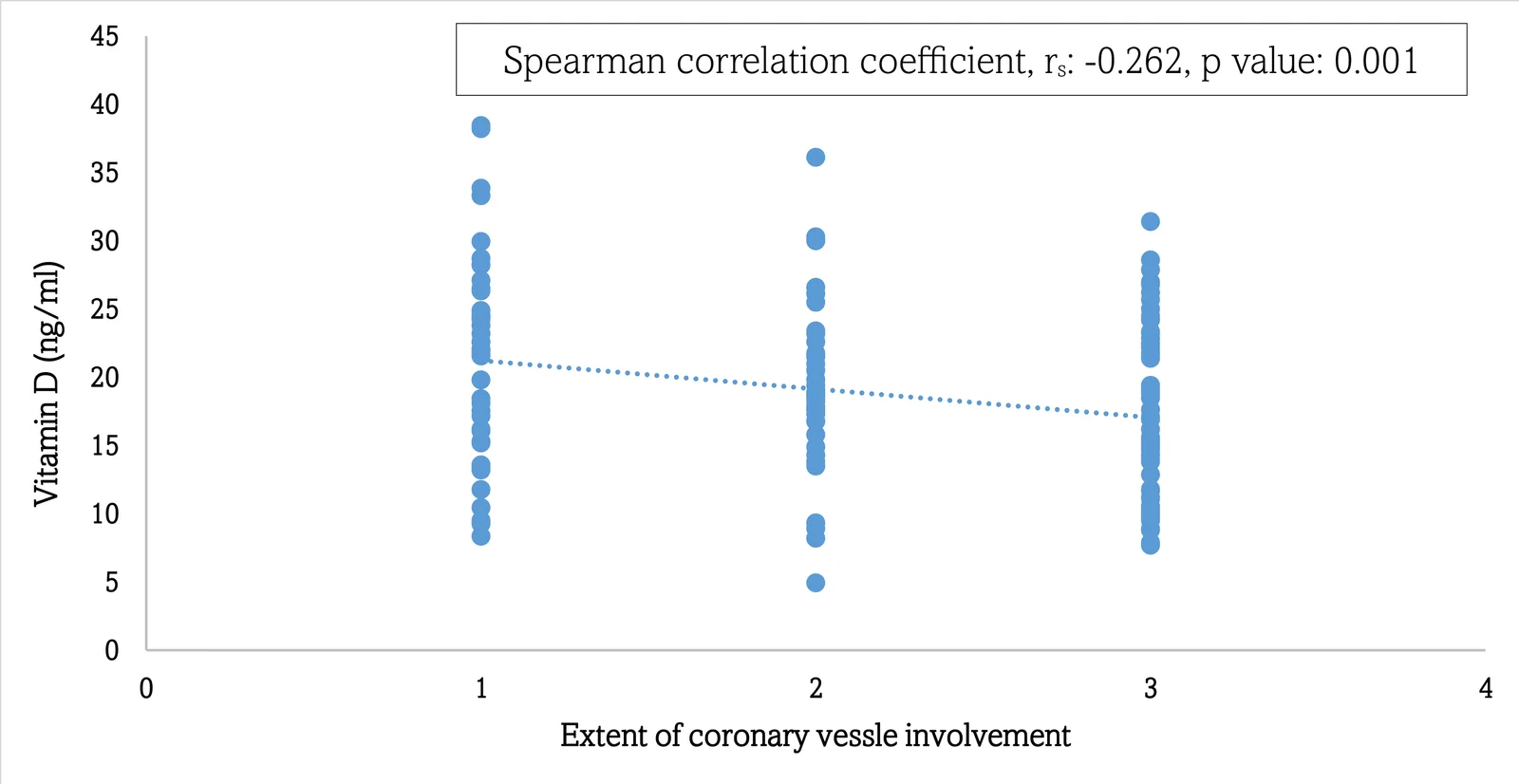
Correlation between serum vitamin D level and extent of coronary vessel involvement among patients with CAD CAD: coronary artery disease

ROC curve analysis showed that serum vitamin D had limited discriminatory ability for distinguishing multivessel from single-vessel CAD, with an AUC of 0.619 (95% CI: 0.513-0.725; p = 0.025) (Figure 3).

**Figure 3:**
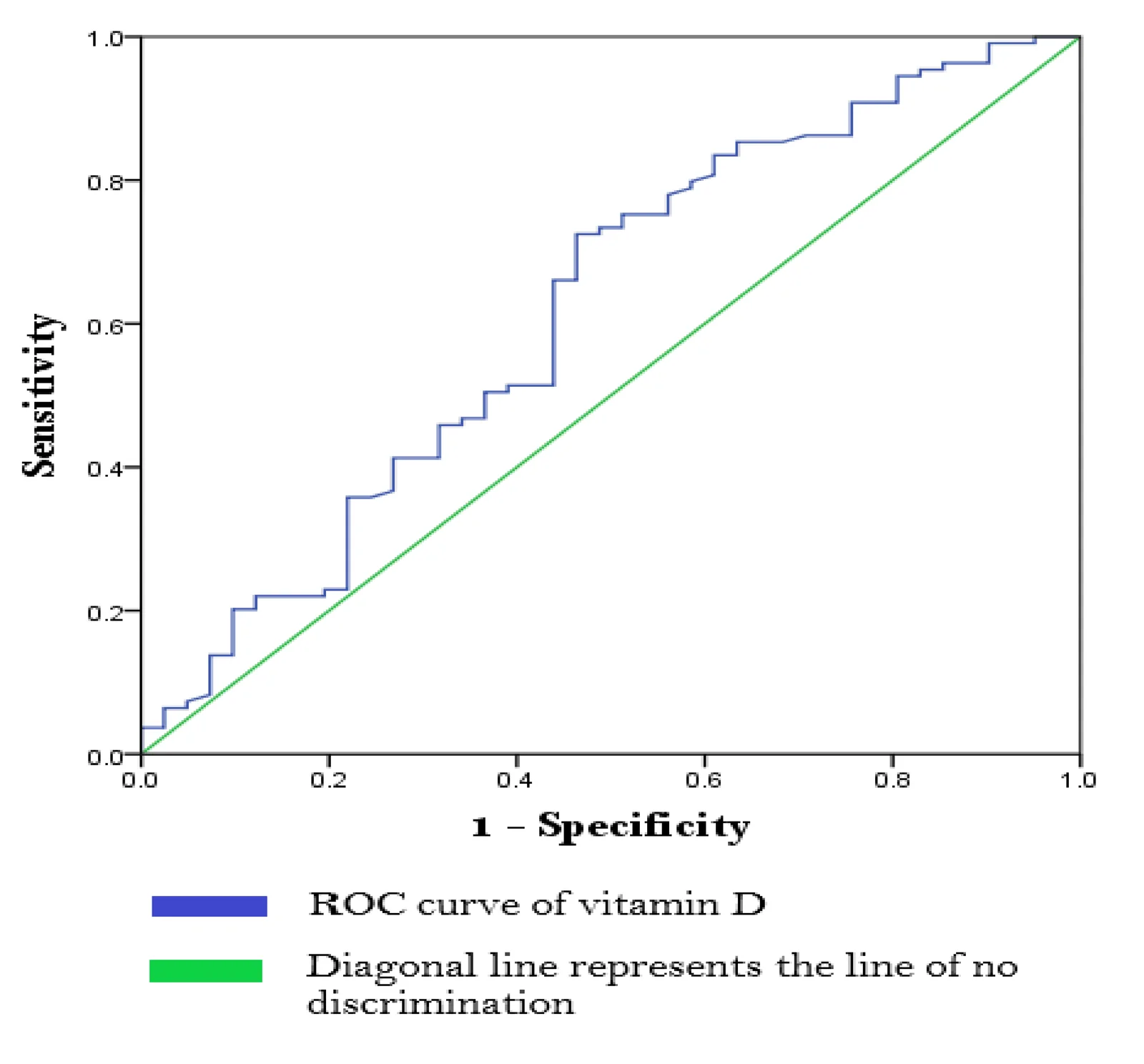
ROC curve of serum vitamin D level for discriminating multivessel from single-vessel CAD ROC: receiver operating characteristic, CAD: coronary artery disease

Using the Youden index, an exploratory serum vitamin D threshold of ≤21.54 ng/mL was identified for distinguishing multivessel from single-vessel CAD. At this threshold, sensitivity was 72.5%, specificity was 53.7%, and overall accuracy was 67.3%. The positive predictive value was 80.6%, while the negative predictive value was 42.3% (Table 4).

**Table 4:** Determination of serum vitamin D threshold for discriminating multivessel from single-vessel CAD using the Youden index CAD: coronary artery disease

Threshold	Sensitivity	Specificity	Positive predictive value	Negative predictive value	Accuracy	Youden index
≤18.30	51.4%	61.0%	77.8%	32.1%	54.0%	0.1235
≤19.61	66.1%	56.1%	80.0%	38.3%	63.3%	0.2215
≤21.54	72.5%	53.7%	80.6%	42.3%	67.3%	0.2614
≤21.69	73.4%	51.2%	80.0%	42.0%	67.3%	0.2461
≤21.91	75.2%	48.8%	79.6%	42.6%	68.0%	0.2401

At the identified serum vitamin D threshold of ≤21.54 ng/mL, discrimination between multivessel and single-vessel CAD yielded a sensitivity of 72.48% (95% CI: 63.10-80.60%), a specificity of 53.66% (95% CI: 37.42-69.34%), and an overall accuracy of 67.33% (Table 5).

**Table 5:** Diagnostic performance metrics with 95% CIs for the identified serum vitamin D threshold in discriminating multivessel CAD CIs: confidence intervals, CAD: coronary artery disease

Statistic	Value	(95% CI)	
		Lower	Upper
Sensitivity	72.48%	63.10%	80.60%
Specificity	53.66%	37.42%	69.34%
Positive predictive value	80.61%	74.57%	85.50%
Negative predictive value	42.31%	32.59%	52.66%
Accuracy	67.33%	59.21%	74.76%

Comparison of participants with single-vessel and multivessel CAD showed that age <50 years, obesity, and serum vitamin D levels ≤21.54 ng/mL were significantly associated with multivessel involvement. In contrast, gender, hypertension, diabetes mellitus, smoking, lipid parameters, HbA1c, and calcium-PTH status did not differ significantly between the two groups (Table 6).

**Table 6:** Association of sociodemographic, clinical, and laboratory characteristics with the severity of CAD ^a^Chi-square test; ^b^Fisher’s exact test. Data are presented as frequencies (percentage). HDL: high-density lipoprotein, LDL: low-density lipoprotein, CAD: coronary artery disease, HbA1c: glycated hemoglobin, ALP: alkaline phosphatase, PTH: parathyroid hormone

Variable		Single-vessel disease	Multivessel disease	p-value
		n = 41	n = 109	
Age (years)	<50	6 (11.8)	45 (88.2)	0.002^a^
	≥50	35 (35.4)	64 (64.6)	
Gender	Male	28 (24.6)	86 (75.4)	0.175^a ^
	Female	13 (36.1)	23 (63.9)	
Hypertension	Yes	16 (29.1)	39 (70.9)	0.713^a ^
	No	25 (26.3)	70 (73.7)	
Diabetes mellitus	Yes	15 (33.3)	30 (66.7)	0.280^a^
	No	26 (24.8)	79 (75.2)	
Obesity	Yes	1 (5.6)	17 (94.4)	0.026^b ^
	No	40 (30.3)	92 (69.7)	
Smoking	Yes	4 (20.0)	16 (80.0)	0.429^a ^
	No	37 (28.5)	93 (71.5)	
Family history of CAD	Yes	4 (33.3)	8 (66.7)	0.736^b ^
	No	37 (26.8)	101 (73.2)	
Vitamin D (ng/mL)	≤21.54	19 (19.4)	79 (80.6)	0.003^a^
	>21.54	22 (42.3)	30 (57.7)	
Total cholesterol	Normal	11 (28.2)	28 (71.8)	0.887^a^
	High	30 (27.0)	81 (73.0)	
HDL	Normal	0 (0.0)	0 (0.0)	-
	Low	41 (27.3)	109 (72.7)	
LDL	Normal	14 (30.4)	32 (69.6)	0.571^a^
	High	27 (26.0)	77 (74.0)	
Triglyceride	Normal	0 (0.0)	3 (100.0)	0.562^b^
	High	41 (27.9)	106 (72.1)	
HbA1c	Normal	21 (27.6)	55 (72.4)	0.934^a^
	High	20 (27.0)	54 (73.0)	
ALP	Normal	39 (26.9)	106 (73.1)	0.614^b^
	High	2 (40.0)	3 (60.0)	
PTH	Normal	21 (28.8)	52 (71.2)	0.701^a^
	High	20 (26.0)	57 (74.0)	
Calcium	Normal	38 (27.9)	98 (72.1)	0.759^b ^
	Low	3 (21.4)	11 (78.6)	

In univariable logistic regression, age <50 years and serum vitamin D level ≤21.54 ng/mL were significantly associated with multivessel CAD (OR 4.102, p = 0.003 and OR 3.049, p = 0.003, respectively). Obesity was associated with higher odds of multivessel disease, although the association did not reach statistical significance (OR 7.391, p = 0.056). In the multivariable model, both age <50 years and serum vitamin D level ≤21.54 ng/mL remained independently associated with multivessel CAD. Participants aged <50 years had 3.614-fold higher adjusted odds of multivessel disease than those aged ≥50 years (AOR 3.614, 95% CI: 1.380-9.467; p = 0.009). Similarly, participants with serum vitamin D levels ≤21.54 ng/mL had 2.669-fold higher adjusted odds of multivessel disease than those with levels >21.54 ng/mL (AOR, 2.669; 95% CI, 1.241-5.741; p = 0.012). Thus, younger age and lower serum vitamin D levels remained independently associated with multivessel CAD within the specified multivariable model (Table 7).

**Table 7:** Univariable and multivariable logistic regression analyses of factors associated with multivessel coronary artery involvement Univariable and multivariable logistic regression analyses were performed. Data are presented as ORs and 95% CIs. Age, vitamin D level, and obesity were assessed in univariable logistic regression. Variables significant in the univariable analysis (age <50 years and vitamin D ≤21.54 ng/mL) were entered into the multivariable model. Accordingly, estimates were adjusted for age and vitamin D level. Obesity was not included in the multivariable model because it did not reach statistical significance in univariable analysis (p = 0.056). ORs: odds ratios, CIs: confidence intervals

Variable	Unadjusted OR	95% CI		p-value	Adjusted OR	95% CI		p-value
		Lower	Upper			Lower	upper	
Age (<50 years)	4.102	1.592	10.566	0.003	3.614	1.380	9.467	0.009
Vitamin D (≤21.54 ng/mL)	3.049	1.449	6.416	0.003	2.669	1.241	5.741	0.012
Obesity	7.391	0.951	57.459	0.056	-	-	-	-

## Discussion

The current study demonstrated a significant inverse association between serum vitamin D levels and the angiographic severity of CAD. Participants with CAD had significantly lower mean serum vitamin D levels than those without CAD. Furthermore, lower serum vitamin D levels and younger age were independently associated with multivessel CAD. Overall, these findings indicate that lower serum vitamin D levels were associated with both the presence of CAD and greater coronary vessel involvement.

In this study, age distribution was comparable between the CAD and non-CAD groups, whereas male sex and higher BMI were significantly more common among participants with CAD. These findings are consistent with previous reports showing a greater occurrence of CAD among men and an association between higher BMI and CAD [18,19]. Participants with CAD also exhibited a less favorable metabolic and biochemical profile, characterized by significantly higher total cholesterol, triglycerides, LDL cholesterol, HbA1c, and PTH levels, together with lower HDL cholesterol, serum vitamin D, and calcium levels compared with those without CAD. The lower serum vitamin D levels observed among participants with CAD are consistent with the findings of Chowdhury et al. and Muvva Venkateswarlu et al. [20,21]. VDD was also more prevalent among participants with CAD, consistent with the high burden of hypovitaminosis D reported in South Asian populations [22].

In this study, STEMI was the most common clinical presentation among participants with CAD, accounting for 42.7% of cases. Previous evidence suggests that serum vitamin D concentrations may be influenced by the acute inflammatory response associated with myocardial infarction. Therefore, because vitamin D was measured during the same clinical episode, an acute-phase influence on the observed vitamin D levels cannot be excluded. Participants with CAD also exhibited several metabolic and biochemical abnormalities, including dyslipidemia, higher HbA1c and PTH levels, and lower calcium levels. Previous studies have proposed biological links between VDD and secondary hyperparathyroidism, insulin resistance, altered lipid metabolism, endothelial dysfunction, and vascular inflammation [22-24]. Dyslipidemia, poor glycemic control, and disturbances in vitamin D-calcium-PTH homeostasis have also been implicated in pathways related to oxidative stress, endothelial injury, and atherosclerosis. However, these mechanisms were not directly evaluated in the present study, and the observed metabolic abnormalities cannot be assumed to result from VDD. Reverse causation and residual confounding cannot be excluded; therefore, these findings should be interpreted as associations rather than evidence that VDD directly contributes to the development or progression of multivessel CAD.

In evaluating the relationship between serum vitamin D levels and the angiographic extent of CAD, serum vitamin D levels decreased with increasing coronary vessel involvement. A weak but statistically significant inverse correlation was observed between serum vitamin D levels and the extent of coronary vessel involvement (rₛ = −0.262, p = 0.001), indicating that lower vitamin D levels were associated with greater coronary vessel involvement. Siddiki et al. similarly reported a moderate inverse correlation between serum vitamin D levels and the Gensini score (r = −0.479, p = 0.001) [25]. Naveed et al. observed a stronger inverse correlation with the Gensini score (rₛ = −0.79), whereas Sahani and Gupta reported inverse correlations with both the Gensini score (rₛ = −0.572) and the SYNTAX score (rₛ = −0.787) [11,26]. In contrast, Muvva Venkateswarlu et al. and Alsancak et al. found no significant correlation between serum vitamin D levels and increasing CAD severity [12,20].

The differences in the magnitude of association across studies should be interpreted cautiously because the methods used to assess angiographic severity were not directly comparable. The present study assessed disease extent according to the number of major coronary vessels involved. In contrast, studies by Naveed et al. and Sahani and Gupta used the Gensini and SYNTAX scoring systems, which incorporate additional information on lesion location, degree of stenosis, and anatomical complexity. Differences in baseline cardiovascular risk profiles, nutritional status, sunlight exposure, prevalence of VDD, ethnic and geographic characteristics, laboratory methods, vitamin D thresholds, and adjustment for potential confounders may also have contributed to the variation in reported correlations across study populations.

In the present study, serum vitamin D demonstrated limited ability to distinguish multivessel from single-vessel CAD, with an AUC of 0.619 (95% CI: 0.513-0.725; p = 0.025). At the identified serum vitamin D threshold of ≤21.54 ng/mL, sensitivity was 72.5%, specificity was 53.7%, and overall accuracy was 67.3%. Ebaid et al. reported a substantially higher sensitivity (98.86%) of serum vitamin D for CAD severity [27]. Candemir et al. identified a vitamin D level of 13.87 ng/mL as being associated with higher SYNTAX scores among obese patients [28]. Differences in study populations, definitions of disease severity, vitamin D thresholds, and analytical approaches may account for the variation in reported performance. Importantly, the threshold identified in the present study was derived and evaluated within the same study population without internal or external validation; therefore, its discriminatory performance should be considered sample-specific and requires validation in independent populations.

In this study, age <50 years and serum vitamin D level ≤21.54 ng/mL remained independently associated with multivessel CAD. Similar findings regarding vitamin D have been reported in an Egyptian study involving 188 patients with CAD and 131 participants without CAD, in which lower vitamin D levels were associated with both the presence and severity of CAD, and multivessel involvement was observed in 60.6% of vitamin D-deficient patients, with a reported 4.43-fold higher likelihood of multivessel disease [29]. The association between age <50 years and multivessel CAD in the present study should, however, be interpreted with caution, as advancing age is generally associated with a greater cumulative atherosclerotic burden. Because the present analysis was restricted to participants who already had angiographically confirmed CAD, younger individuals presenting with established disease may represent a selected subgroup with premature CAD [30]. Selection characteristics, residual confounding, and sampling variability may also have contributed to this finding. Accordingly, neither younger age nor lower vitamin D level should be interpreted as establishing a causal determinant of multivessel CAD based on the present observational data.

Limitations

Several limitations should be considered when interpreting the findings of this study. First, the hospital-based cross-sectional comparative design does not permit assessment of temporal or causal relationships between serum vitamin D status and the extent of CAD. Additionally, participants were recruited from two tertiary care hospitals among patients undergoing clinically indicated coronary angiography, and the non-CAD group consisted of symptomatic patients without angiographic evidence of CAD rather than healthy individuals. Therefore, the findings may not be generalizable to the general population. Second, serum vitamin D was measured at a single time point, while seasonal variation, sunlight exposure, dietary habits, physical activity, and socioeconomic factors were not comprehensively assessed. A substantial proportion of participants with CAD presented with acute myocardial infarction, including STEMI, and vitamin D was measured during the same clinical episode. Thus, an acute-phase influence on serum vitamin D concentration and residual confounding related to clinical presentation cannot be excluded. Third, residual confounding and reverse causation remain possible because factors such as duration, control of comorbidities, medication use, and inflammatory status were not fully adjusted for. Accordingly, the direction of the observed association between lower vitamin D levels and more extensive CAD cannot be determined. Fourth, the serum vitamin D threshold of ≤21.54 ng/mL was identified and evaluated within the same study population without internal or external validation. Its discriminatory performance should therefore be considered sample-specific and requires validation in independent populations before clinical application. Fifth, angiographic severity was assessed by the number of coronary vessels involved rather than by more comprehensive scoring systems such as the Gensini or SYNTAX score, which may have provided a more detailed assessment of coronary lesion severity and anatomical complexity.

## Conclusions

The present study identified a significant association between lower serum vitamin D levels and greater angiographic severity of CAD. Participants with CAD had significantly lower serum vitamin D concentrations than those without angiographic evidence of CAD, and serum vitamin D showed a weak but statistically significant inverse correlation with the number of coronary vessels involved. Within the specified multivariable model, age <50 years and serum vitamin D level ≤21.54 ng/mL remained independently associated with multivessel CAD. However, the discriminatory ability of serum vitamin D for multivessel CAD was limited, and the identified threshold was derived from the same study population. Therefore, serum vitamin D should not be considered a stand-alone marker of CAD severity based on the present findings. The findings suggest a potential role for serum vitamin D as an adjunctive biomarker of more extensive coronary involvement; however, its clinical utility and discriminatory ability require further evaluation in larger prospective studies with comprehensive adjustment for relevant confounders and independent validation.
